# Influenza preparedness and response: Involvement of African Field Epidemiology and Laboratory Training Programs, 2009

**Published:** 2011-09-28

**Authors:** Nykiconia Preacely, Peter Nsubuga

**Affiliations:** 1 US Centers for Disease Control and Prevention, Division of Public Health Systems and Workforce Development, Atlanta, GA, USA

**Keywords:** Influenza, FELTP, surveillance, preparedness, response, H1N1, pandemic, field epidemiology

## Abstract

**Background:**

The capacity of public health professionals to rapidly detect and respond to disease pandemics is critical to understand and control global disease spread. On June 11, 2009, the World Health Organization (WHO) declared H1N1 virus infection as pandemic. In May 2009, we assessed the participation of Field Epidemiology and Laboratory Training Programs (FELTPs) based in sub-Saharan Africa on pandemic influenza preparedness and response.

**Methods:**

We administered an electronic survey to directors and resident advisors of African Field Epidemiology Network (AFENET) member and associate FELTPs. The survey included questions on the following attributes: program involvement in suspected H1N1 investigations, experience in influenza outbreak investigations, national influenza surveillance and response plans, and H1N1 outbreak preparedness.

**Results:**

Nine countries (100%) responded to the survey; all had existing national influenza response plans. Six programs reported their trainees had participated in past pandemic preparedness and response exercise, five (83%) of them were influenza specific.

**Conclusion:**

FELTPs played an important role in H1N1 surveillance and response in sub-Saharan Africa. Continued technical assistance and support to these programs is vital to foster their capacity to monitor and control public health threats.

## Background

The initial response to an infectious disease outbreak is a public health function of a domestic government [1]. The competence of public health professionals to rapidly detect and respond to disease pandemics is critical to understand and control global disease spread. In sub-Saharan Africa there is an ongoing need to staff Ministries of Health (MoH) with individuals well trained in public health preparedness to serve as in-country first responders. MoH public health workforce shortages may be attributed to a number of factors including a lack of public health training programs, financial resource constraints, political instability, and competition with outside agencies. In addition to a loss of workforce to domestic opportunities outside of the MoH, developing countries with highly trained public health workers often experience problems retaining them and are forced to compete with well established international agencies and nongovernmental organizations (NGOs) in higher income countries. In the mid-1990s Public Health School Without Walls (PHSWOW) were established in Zimbabwe and Uganda to link ministries of health with universities providing two year master's degree training on management, economics, and epidemiology [2]. The first Field Epidemiology and Laboratory Training Program (FELTP), a two year competency-based program in applied epidemiology and laboratory practice, was created in 2004 in Kenya as a regional program to help address the shortage of advanced trained public health professionals in Kenya and neighboring countries [3]. After two years FELTP graduates return to their respective countries to build public health capacity through the development and operation of disease surveillance and response systems amongst other critical public health responsibilities.

In 2005, the African Field Epidemiology Network (AFENET) was formed as a non-profit organization to serve as a networking alliance for FELTPs to support MoH build and sustain effective public health systems in Africa. In addition to the MoH, AFENET works in partnership with several governmental and NGOs within and outside of Africa, including the U.S. Centers for Disease Control and Prevention (CDC), to strengthen public health systems and workforce capacity [4]. The Kenya FELTP has served as a flagship model for subsequent AFENET member and associate FELTPs (AFENET- FELTPs) in Africa (Table 1), all of which have laboratory management components and are associated with a local university that grants a masters degree upon completion of the FELTP. In addition to advanced public health training offered in FELTPs, AFENET reaches a larger audience of host-country public health workers through 2-week short courses that provide fundamental field epidemiology and laboratory trainings to prepare frontline staff operate basic multi-disease surveillance and response systems [5]. In the sections below, we describe an assessment of AFENET- FELTPs involvement in influenza surveillance and response activities.

**Table 1:** African Field Epidemiology Network (AFENET) Affiliated Public Health Schools without Walls (PHSWOW) and Field Epidemiology and Laboratory Training Programs (FELTP) by Country, April-August 2009

Country	Program Type	Year of 1^st^FELTP Cohort	Reported # of FELTP graduates	Reported # of current FELTP trainees
Zimbabwe	PHSWOW	1993	104	42
Uganda	PHSWOW	1994	200	29
Kenya and South Sudan	Regional FELTP	2004	40^a^	27^b^
South Africa	FELTP	2006	10	22
Ghana	FELTP	2007	…^c^	13
Nigeria	FELTP	2008	…^c^	13
Tanzania	FELTP	2008	…^c^	12
Ethiopia	FELTP	2009	…^c^	13
**Total**			**354**	**171**

^a^ Includes 13 graduates from Southern Sudan that were trained in the Kenya program

^b^ Includes 9 trainees from Southern Sudan that were trained in the Kenya program

^c^ All FELTP trainees were in either first or second year of training

## Methods

Prior to the phase 6 pandemic H1N1 influenza alert released by the World Health Organization [6] on June 11, 2009 as a result of widespread global transmission of H1N1, we developed a tool to assess influenza surveillance and response activities of AFENET- FELTPs trainees to provide insight into optimizing their capacity to prepare and respond to a possible influenza pandemic. In early May 2009, an invitation to complete an electronic survey was emailed to the directors and resident advisors of all eight AFENET-FELTPs and to FELTP field supervisors of Kenya trainees supporting public health activities in Southern Sudan. One response per country was sought. Follow-up emails and phone calls were made 4 weeks later to promote timely participation. The survey included questions on the following program attributes:

- Pandemic preparedness and response training
- Influenza outbreak investigation experience
- National influenza surveillance and response planning
- H1N1 outbreak involvement

Descriptive analyses were performed using SAS^TM^ software, Version 9.1.3 [7].

## Results

Nine survey responses were received between June and August 2009, which represented 100% of AFENET-FELTP countries (Zimbabwe, Uganda, Kenya, South Africa, Ghana, Nigeria, Tanzania, Ethiopia) with active programs at the time of the survey, inclusive of one response describing Kenya FELTP resident activities based in Southern Sudan. A total of 171 FELTP trainees were engaged in public health activities at the time of this survey in their respective countries (Table 1). Six (67%) of the countries had organized FELTP rapid response teams. Fourty-four respondents (44%) rated their FELTP trainees as having very good knowledge of disease surveillance and response activities, thirty-three (33%) good knowledge, and twenty-two (22%) average knowledge.

Most of the AFENET-FELTPs were involved in national influenza response plans (Table 2). In six of the nine (67%) countries FELTP trainees were engaged in influenza surveillance activities. In three (33%) countries FELTP trainees were involved in influenza outbreak investigations within the year prior to the 2009 H1N1 pandemic. FELTP trainees in six (67%) countries participated in pandemic preparedness and response activities during their training, five (83%) of them were influenza specific. Seven (78%) countries reported investigations of H1N1 cases between April and August 2009; FELTP trainees led five (71%) of these investigations. Five (100%) of the FELTPs involved in H1N1 investigations collaborated with the US CDC or local CDC country office on laboratory support.

**Table 2:** Select Qualitative Questions and Responses from an Assessment of African Field Epidemiology Network (AFENET) Affiliated Public Health Schools without Walls (PHSWOW) and Field Epidemiology and Laboratory Training Programs (FELTP) by Country of Trainee Assignment, April-August 2009

**Question- What is your program's involvement in your country's national influenza response plan?**	Field Epidemiology and Laboratory Training Program/ Public Health Schools without Walls Response
	Country 1- Plan development and influenza surveillance
	Country 2- Participation in outbreak investigations teams
	Country 3- Part of ministry of health response as assigned
	Country 4- National focal point on the H1N1 task force, development and implementation of response plan, and mobilization of resources from the AFENET network
	Country 5- None, plan was developed prior to the start of FELTP
	Country 6- Participation in influenza sentinel surveillance
	Country 7- Participation on rapid response team at the central level
	Country 8- Surveillance and contact tracing
	Country 9- Participates on the preparedness committee, national sensitization teams and case investigations
**Question-What preparation is being done by your program to respond to a possible H1N1 outbreak in your country?**	Country 1- Mobilizing resources for influenza training and outbreak response, surveillance activities, and involvement of trainees in zoonotic outbreak investigations
	Country 2- Contributed to establishing and operating surveillance and quarantine facilities at international airport
	Country 3- Preparation of trainings materials for H1N1 surveillance and response, setting up surveillance systems at the major national airports
	Country 4- Managing databases, responding to public inquiries about H1N1, screening potential cases, and providing advice to clinicians and public health authorities
	Country 5- Participation in the national taskforce coordination meetings and conducting influenza sentinel surveillance
	Country 6- Conducting country preparedness surveys and participating in the influenza task force
	Country 7- Stockpiling of personal protective equipment and medications, activation of rapid response teams, and communicating H1N1 health messages on national radio stations
	Country 8- Participating in H1N1 surveillance at country ports of entry and H1N1 contact tracing
	Country 9- Participation in national preparedness committee planning

## Discussion

The emergence and global spread of the novel influenza virus H1N1 in 2009 displayed the critical need for domestic public health professionals prepared to detect and rapidly respond to the pandemic. Many countries in sub-Saharan Africa have inadequate human resource capacities for public health [8] and weak health systems. FELTP and affiliated short course graduates are trained to manage and operate robust multi-disease surveillance systems. FELTP's have proven to be successful in training critical masses of workers [2,9] and strengthening public health infrastructure of MoH in sub-Saharan Africa [10]. Program co-ownership by the MoH and local universities in sub-Saharan Africa is beneficial for worker retention because the FELTP training is catered to the public health needs of the country and it well defines in-country career paths for FELTP graduates [9].

The findings of this assessment show that AFENET-FELTP trainees played frontline roles in local influenza surveillance system development and operation prior to and during the early stage of the 2009 H1N1 pandemic. As rapid responders to disease outbreaks it is important that trainees are equipped with the proper epidemiologic, laboratory, and management expertise to respond to emerging pandemics [5]. An added value of the AFENET-FELTPs is their incorporation of laboratory management specifically focused on integrating surveillance and response activities with laboratory services. Laboratory collaborations with CDC offices assisted FELTP trainees with laboratory capacity to confirm cases of H1N1. An important limitation of this assessment is that it was self-administered and may have led to reporting bias.

## Conclusion

AFENET-FELTPs contribute important human resource capacity to support public health activities in their host countries. However, technical support and funding are challenges for many FELTPs. MoH and other key partner support are needed to build and sustain FELTPs. Since this assessment, four AFENET-FELTPs have begun (Rwanda, established in 2010; West Africa regional, 2010: (Burkina Faso, Mali, Niger, and Togo); Mozambique, 2010; and Central Africa regional, 2010: Cameroon, Central Africa Republic and Democratic Republic of Congo). Continued expansion of FELTPs and short course training in additional sub-Saharan African countries will strengthen the network of first line responders to public health threats in the region.

At the onset of the 2009 H1N1 pandemic, FELTP trainees led H1N1 surveillance and response activities in a majority of the AFENET-FELTP countries. This recent pandemic demonstrates the importance of having local human capacity to detect and respond to a massive public health event. Sustained training opportunities and well-defined career paths are essential for countries in sub-Saharan Africa to retain quality professionals. Continued technical assistance and collaboration with partners is important to help AFENET-FELTPs develop a skilled public health workforce that will in turn strengthen and operate public health systems in these countries.
